# The association of Q472H variant in the *KDR* gene with recurrent pregnancy loss in Southern Iran: A case-control study

**DOI:** 10.18502/ijrm.v17i7.4858

**Published:** 2019-07-31

**Authors:** Leila Keshavarz, Majid Yavarian

**Affiliations:** ^1^Department of Biology, Islamic Azad University, Arsanjan Branch, Arsanjan, Iran.; ^2^Shiraz Nephron-Urology Research Center, Shiraz University of Medical Sciences, Shiraz, Iran.

**Keywords:** Abortion, VEGFR 2, Polymorphism.

## Abstract

**Background:**

Recurrent spontaneous abortion (RSA) often remains unclear and can be burden for the patient and time consuming for clinician. RSA may initiates from a genetic or non-genetic factors. It is well known that the quality of placental circulation is critical for implantation and embryo development. Because of angiogenic effects of VEGF–KDR pathway on placenta, the genes involved in this pathway (the *KDR* or *VEGFR* genes) are thought to be linked with RSA.

**Objective:**

The aim of this study was to investigate the relationship between Gln472His (A/T) polymorphism of the *KDR* gene with RSAs in southern Iran.

**Materials and Methods:**

In this case-control study, 50 aborted embryonic tissue obtained from fetuses and 50 umbilical cord blood of newborn babies were studied. Fetal sample from mothers with history of at least two consecutive miscarriages and controls from mothers who had at least one full-term infants born were taken. Genomic DNA was extracted by using PureLink genomic DNA kit (Life Technologies, CA). The Rotor-Gene Q real-Time PCR machine and High-resolution melting curve analysis (HRM) technique were used for genotyping.

**Results:**

Based on the AA genotype as reference, it is shown that the T allele (OR = 2.447, 95% CI = 1.095–5.468, p = 0.029) as well as AT heterozygote genotype was significantly associated with an increased risk of miscarriage (OR = 2.824, 95% CI = 1.210-6.673, p = 0.016).

**Conclusion:**

A positive correlation between Q472H polymorphism of the *KDR* gene and RSA may be the cause in southern Iran.

## 1. Introduction

Three or more consecutive pregnancy losses before the 20${}^{\mathrm{th}}$ wk of gestation is referred to as recurrent spontaneous abortion (RSA) (1). This is a reproductive issue that challenges approximately 1% of women at the reproductive age, and the causes of about half of the RSA cases remain undecided (2-4). Chromosomal abnormalities are one of the main causes of spontaneous abortion (5) followed by molecular genetic defect like inherited thrombophilia. Non-genetic causes including abnormalities of the uterus, dysfunctions of the endocrine system, autoimmune diseases, as well as nutritional and environmental parameters are also mentioned on the etiology of RSA (6, 7). Vascular endothelial growth factor (VEGF) as a main angiogenic factor has a primary role in the regulation of endothelial cell proliferation. It also acts as a survival factor for endothelial cells during both physiological and tumor angiogenesis. Furthermore, VEGF is a vasodilator that increases vascular permeability and has anti-apoptosis properties (8, 9). VEGF is a critical factor in the early stages of pregnancy such as oocyte maturation and the growth of trophoblast. It is also important in the implantation and subsequent development of the embryo. Moreover, the angiogenesis during placenta development as well as the formation of maternal and fetal blood vessels in the uterus is related to VEGF (10, 11). There are two tyrosine kinase receptors for VEGF, VEGF receptor 1 (VEGFR1/FLT1) and VEGF receptor 2 (VEGFR2/FLK1/KDR) (12). These two VEGF receptors -a signal-transducing receptor- carries Kinase domain, which has also been stated to be associated with RSA in report, possibly resulting from the effects on the angiogenesis of placenta via the VEGF-KDR pathway (11, 13, 14). Placental angiogenesis is important for the successful development of a fetus considering the influences on the transplacental exchange between maternal and fetal blood flow (15). In human during the early pregnancy, the *KDR* gene expresses has critical roles on normal physiology or physiopathology of placental precursor cells. It has influence on the vasculogenic as well as angiogenic of proliferative endothelial cells and altered quality of its migration as well (16, 17). Experimentally in the *KDR* gene knockout, mice embryonic death occurs due to defects in the fetal and placental vasculogenesis and angiogenesis (18). A number of single nucleotide polymorphisms (SNPs) in the *KDR* gene have been reported that have effect on the pathogenicity of several diseases including RSA, lung and breast cancers, coronary heart disease, as well as cerebrovascular disorder (19). These SNPs include –604T/C at the KDR promoter region alternate/affect promoter activity, 1192G/A (rs2305948) in exon 7 with the Val297Ile substitution, and 1719A/T (rs1870377) in exon 11 with the Gln472His substitution effects on the function of VEGF/KDR pathway (20, 21).

In Iran, there are at least two studies regarding the *KDR* gene polymorphism and RSA (22, 23). These reports have been done on the mother's blood and the effect of paternal imprint is mistreated. In this study, the 1719A/T in the exon 11 of *KDR* gene of fetal as a risk factor for RSA in our area has been analyzed.

## 2. Materials and Methods

### Subjects

In total, 50 samples of embryonic tissue (obtained from fetuses) and 50 umbilical cord blood of newborn babies were selected randomly from the Shiraz Hafez Hospital (Jan-May 2017). The fetus selected from mothers who had experienced at least two consecutive miscarriages earlier and controls from mothers had at least one full-term infants. These groups Maternal control group and cases group were matched by ethnicity and age. All subjects with underlying diseases or abnormal laboratory data including immunologic, infection, or abnormal karyotype were excluded from the controls.

### Genetic analysis

Genomic DNA from embryonic tissue as well as whole EDTA blood was extracted using PureLink genomic DNA kit (Life Technologies, CA) according to the manufacturer instructions. The quantity and quality of extracted DNA were checked by Nanodrop spectrophotometer (BioTek company) and 0.8% agarose gel electrophoresis, respectively. All DNA samples were stored at -20°C until further analysis. Genetic variations for Gln472His (A/T) SNP was analyzed by LC-Green (Idaho Technology, Salt Lake City, Utah, USA) high-resolution melting (HRM) curve method following the PCR amplification (Qiagen company).

### Primers sequences

Sequence of used primers for Gln472His (A/T) polymorphism genotyping is:

Forward primer: (5'-GGAAGTCCTCCACACTTCTCC-3')

Reverse primer: (5'-GTACCATGGTAGGCTGCGTT-3')

### PCR reactions and melting analysis

PCR was performed in 8 μl volum with 20 ng of template DNA, 1.5 mM MgCl2, 265 μM dNTP, 10 pico mol of forward and reverse primers, and 0.8X LC-Green Plus (Idaho Technology, Salt Lake City, Utah, USA). Platinum Taq Polymerase (Invitrogen, Paisley, Scotland) is used for PCR.

PCR cycling conditions included an initial activation step at 95°C for 5 min followed by 40 cycles of denaturation for 10 s at 95°C and annealing/extension step for 30 s at 55°C to allow for fluorescence data acquisition on the green channel. A 192 bp PCR product is accomplished by HRM analysis in the range of 65-95°C and 0.1°C rising at each cycle. All test run with null template control (NTC) and control of known genotype on each course. A Sanger sequence confirmed sample is used as control in HRM-Realtime PCR and 20 samples (case and control) were randomly selected and checked by sequencing for re-conformation.

### Ethical consideration

The research was performed whit the approval of the Ethics Committee of the Islamic Azad University, Dehaghan Branch (Approval No. IR.IAU.DEHAGAN.REC.1397.002), a written consent was obtained from all the mother whose fetuses or cord bloods were used.

### Statistical analysis

Statistical analysis performed by using SPSS software version 18.0 statistical program (Statistical Package for the Social Sciences, SPSS Inc., Chicago, Illinois, USA). Chi-square test and logistic regression analysis were used to analyze the differences between the abortion materials and control subjects. Corresponding genotype and allele frequencies between fetal cases and control group is compered. For the risk assessment of the KDR SNP, Odds ratio (OR) and p-values were used.

## 3. Results

**Figure 1:**
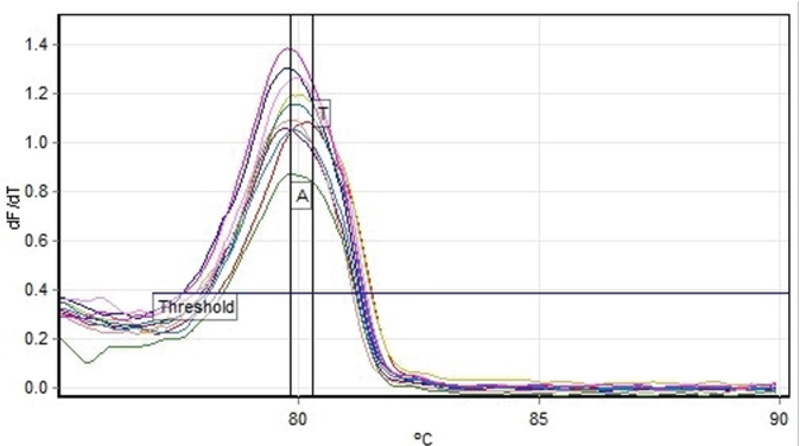
HRM profiles of the product 10–192 bp of the *KDR* Gln472His polymorphism.

Real-time PCR and Melting Analysis technique, Gln472His (A/T) polymorphism of the *KDR* gene were evaluated in study groups (50 abortion materials and 50 control subjects) (Figure 1), and the results were compared between both the fetus and control groups. The mean age of the mothers of case group was 32.07 ± 4.23 yr, and the mean age of the mothers of controls was 33.49 ± 3.80 yr. The other diseases that may contribute with pregnancy outcome exclude in the study groups. Demographic characteristics and clinical profiles of RSA patients and controls are summarized in Table I for comparison. For the controls and RSA patients, genotype and allele frequencies of polymorphism KDR Gln472His (A/T) are listed in Table II. All genotype distributions among the study patients were in Hardy–Weinberg equilibrium. In the statistical analysis, AT genotype and T-allele in dominance were found as risk factors for abortion in case group. AT genotype represents and T-allele in dominance represents 2.824 and 2.447-fold risk reactively in fetus group compared with the control group. The difference in genotypes frequencies between fetus and control group was not statistically significant (p > 0.05) (Table II).

**Table 1:** Demographic data of cases and controls


**Characteristic**	**Case group (n = 50)**	**Control group (n = 50)**	p-value*
Range of mother age	26–39	25-38	–
Average of mother age	32.77 ± 4.32	32.59 ± 3.89	0.942
Average of fetus age, mean wk ± SD	12.05 ± 3.2	–	–
Average age of infants born, mean wk ± SD	–	38.54 ± 1.16	–
Range number of abortions	2-4	–	–
Average number of abortions	3.05 ± 1.64	–	–
Note: Data are mean ± SD unless otherwise specified			
*P-value by Fisher's exact test; otherwise by χ${}^{2}$-test			

**Table 2:** Genotype frequencies of Gln472His polymorphism of the *KDR* gene among controls and RSA patients


**Allele/Genotype**	**Case group**	**Control group**	**p-value**	**OR**	**95%CI**
AA AT TT	30 (60%) 15 (30%) 5 (10%)	19 (38%) 27 (54%) 4 (8%)	– 0.016 0.750	– 2.824 1.631	Reference 1.210-6.673 0.301-5.304
A T	75 (75%) 25 (25%)	65 (65%) 35 (35%)	– 0.124	– 1.615	Reference 0.877-2.977
AT + TT (T+)	20 (40%)	31 (62%)	0.029	2.447	1.095-468

## 4. Discussion

Far province is inhabitancy of multi-ethnic group with distinct genetic property. Genetic variance play a crucial role on the cause of morbidity and mortality of fetus.

The implantation of the embryo is a vital stage in reproduction. It requires an appropriate communication between the mother as a receptive endometrium and the embryo. Various factors affect endometrium which can be summarized as follows various cytokines, growth factors, and adhesion molecules (24-26). Among these factors, VEGF and its receptors, VEGF receptor 1 (VEGFR1/ Flt1) and VEGF receptor 2 (VEGFR2/Flk1/KDR) have a very important role in the formation of the cardiovascular system (27). Other investigations have also reported that disturbances in the vascular formation and function might be the contributing factor in several female reproductive disorders, for example, recurrent miscarriage and implantation failure (12, 28, 29). The necessity of angiogenesis in a successful pregnancy and RSA through VEGF and one of its receptors, KDR, has been proposed in multiple studies (11, 15, 30, 31). A report from Iran show that Q472H (rs1870377) was highly linked to intronic SNP (rs6838752) (23). We surveyed whether KDR polymorphisms, including the Gln472His (A/T) SNP is a risk factor for RSA in southern Iran. The aim of this study was to evaluate KDR polymorphisms that have been previously studied for the association with RSA risk. Our findings reveal that the KDR Gln472His (A/T) polymorphism is consistently associated with increased RSA risk and the allele A as the reference allele shows significant differences between the case and control samples (p = 0.016). Present data are in agreement with association of Q472H and A-C-A-T-G haplotype in the *KDR* gene with susceptibility to miscarriage (11). Binding of VEGF to KDR triggers multiple downstream signaling cascades, including PI3 kinase-Akt, protein kinase C, mitogen-activated protein kinases, and eNOS pathways, and induce migration and proliferation of endothelial cells. Consequently, this stimulation proceeds angiogenesis (32). It is believed that the mentioned polymorphisms altered the biological activity of corresponded pathway (21, 33, 34). The position of Q472H polymorphism, regarding the phenotypic condition in the favor of the dominance of the T allele and this allele increases the risk of abortion by 2.4 times. The Q472H SNP (rs1870377), is located in the exon 11 and covers the fifth N-terminal Ig-like domains within the extracellular region. The extracellular domain is essential for ligand binding. The substituted amino acid at the codon 472 reduc VEGF-binding efficiency of tyrosine receptor (KDR) (21). During embryonic development, the KDR expression is restricted to endothelial cells and encoded protein has a crucial role on endothelial growth. precursors. Studies in KDR knockout mice shows its vital roles in the developing of blood vessel networks (14, 18, 35). Also it is shown, this substitution of Glutamine by Histidine increases VEGFR-2 protein phosphorylation thereafter micro vessel density increases among lung cancer cases (36).

However there some reports that contraindicate with this finding like study by Rah *et al*. who did not find the KDR polymorphism susceptibility to RSA in Taiwanese as wel as Korean cases. (20, 37). Contradictory results between two Asiatic ethnic groups, may arise from various factors, including a different definition of patients and controls for each study and ethnic variation or a combination of them. However, the limited sample size in this study may be required to replicate with a larger sample size. Fine mapping of specific loci within VEGF/KDR and extra genic variants will be the future direction and will provide more information on the functional SNPs in these two genes.

## 5. Conclusion

There is a significant correlation between Q472H polymorphism and related polymorphism in the *KDR* gene with the risk of RSA and it should take into account the RSA cases in the south of Iran.

##  Conflict of Interest

There is no conflict of interest in this article.
